# National Society of Genetic Counselors' Board of Directors response to Myers et al. ‘A report of the AGCPD task force to evaluate associations between select admissions requirements, demographics, and performance on ABGC certification examination’

**DOI:** 10.1002/jgc4.1569

**Published:** 2022-03-08

**Authors:** Heather Zierhut, Leila Jamal, Sara Riordan, Patrick Wilson, Meghan Carey, Deepti Babu

**Affiliations:** ^1^ Department of Genetics, Cell Biology, & Development University of Minnesota ‐ Twin Cities Minneapolis Minnesota USA; ^2^ Department of Health, Behavior, and Society Johns Hopkins Bloomberg School of Public Health Baltimore Maryland USA; ^3^ Unified Patient Network Charlestown Massachusetts USA; ^4^ College of Medicine University of Oklahoma Health Sciences Center Oklahoma City Oklahoma USA; ^5^ National Society of Genetic Counselors Chicago Illinois USA; ^6^ Integrity Content Consulting St. Albert Alberta Canada

In a recent original research article in this journal, Myers et al. reported findings supporting the widely held assumption that white female trainees have higher odds of passing the American Board of Genetic Counseling (ABGC) certification examination on their first attempt than their non‐female non‐white peers (Myers et al., 2021). These findings raise questions about the pitfalls of using standardized testing as the primary measure of preparedness to enter genetic counseling practice. The Myers et al. paper also invites a broader conversation about how our profession's current professional practice standards may pose an obstacle to build a more just, equitable, diverse, and inclusive genetic counseling workforce. We applaud the Association of Genetic Counseling Program Directors (AGCPD) task force for bringing these issues to attention at a time when our profession is working hard to remedy ways that structural, systemic, cultural, and historic forces have combined to result in a poor representation of non‐white non‐female‐identifying people among our ranks.

The goal of this response is to articulate the perspectives of the National Society of Genetic Counselors’ (NSGC’s) Board of Directors on these issues, focusing on the relationship between evolving practice standards for genetic counselors and our fervent wish to build a more diverse, equitable, and inclusive profession. We begin with a reference to how genetic counseling practice standards are currently set and existing gaps in the standards. Subsequently, we emphasize relevant history and action items resulting from two significant professional initiatives: an effort to define genetic counseling outcomes and develop rigorous genetic counseling metrics; and a comprehensive diversity, equity, and inclusion (DEI) assessment for NSGC, which offers a detailed exploration of common barriers and potential new pathways to the genetic counseling profession. Finally, we share our vision for how North American genetic counseling professional organizations might work together to realize the interrelated goals of professional excellence and social justice.

## 1 GENETIC COUNSELING PRACTICE STANDARDS

Despite a growing body of research on genetic counseling processes and outcomes, there are few consensus‐adopted, valid, replicable measures of processes and outcomes of high‐quality genetic counseling (Athens et al., 2017; Cragun & Zierhut, 2018; Kasparian et al., 2007; Madlensky et al., 2017; Meiser et al., 2008; Redlinger‐Grosse et al., 2017, 2020). This makes it challenging to develop a single, universal assessment of competence to enter genetic counseling practice. Ideally, an examination measuring genetic counseling competence would align with definable and measurable skills associated with desirable genetic counseling outcomes, such as patient understanding, decision‐making, and empowerment (Grant et al., 2019; Joseph et al., 2019; Kasparian et al., 2007; Redlinger‐Grosse et al., 2020).

Currently, the Accreditation Council for Genetic Counseling (ACGC) sets standards for training program accreditation and curriculum development. The ACGC’s practice‐based competencies (PBCs) are used as a foundation for training program curricula and preparation for the ABGC certification examination. The certification examination is based on an ABGC practice analysis of currently employed genetic counselors. The practice analysis defines the job activities and responsibilities in which knowledge, skills, and expertise are required for competence in genetic counseling. The certification examination is used as a requirement for licensure in all US states licensing genetic counselors (*States Issuing Licenses*, n.d.). While the PBCs speak broadly to areas in which genetic counselors should demonstrate competence and the examination assesses competence based on the practice analysis, neither are focused on outcomes, nor are they sufficiently specific or comprehensive regarding the skills and expertise that genetic counselors should demonstrate in the wide range of roles they occupy today. If our profession is to gain broader recognition for bringing about desired outcomes, we need to align training competencies with desired outcomes in an evidence‐based way. As such, a more specific and granular framework for training competencies and subsequent measurement of the genetic counseling content knowledge, expertise, and skills associated with improved outcomes is needed. To our knowledge, these issues have been considered, but a new approach has not yet been implemented.

In 2019, in an effort to advance scholarship on genetic counseling processes and outcomes, NSGC began to develop an infrastructure to accelerate research. These efforts focused on defining and measuring psychological, behavioral, medical, interpersonal, and economic outcomes of genetic counseling as well as strategies and approaches that could ultimately lead to better health outcomes (Senter et al., 2020). A related landscape analysis of genetic counseling research conducted in 2020 by Discern Health (unpublished, internal report to NSGC Board of Directors) has identified that a long‐term commitment and collaborative research environment is needed to develop rigorous genetic counseling metrics. Genetic counseling metrics, such as those testing outcomes of evidence‐based patient care, could also be used to investigate factors and variables associated with passing the ABGC examination. The field, therefore, has the potential to work together on inclusive definitions of genetic counselor competence, quality, and prioritization of outcomes beyond the patient's interaction with genetic counselors, and to find ways to measure the most important and impactful ones (Redlinger‐Grosse et al., 2020; Southwick et al., 2020). Of particular importance is recognizing that as the field is predominantly white female‐identifying genetic counselors working with increasingly diverse patient populations, continued efforts are needed to diversify the profession, research participation, and expand genetic services to effectively serve a broader population of patients and consumers.

## 2 A BRIEF HISTORY OF NSGC’S APPROACH: FROM CULTURAL COMPETENCE TO JUSTICE, EQUITY, DIVERSITY, AND INCLUSION

Since its inception in 1979, NSGC has recognized the need to advocate for DEI, both for the populations that genetic counselors serve and within the profession itself. These efforts have evolved over the years, with a timeline of activities available on the NSGC Web site: https://www.nsgc.org/Policy‐Research‐and‐Publications/Justice‐Equity‐Diversity‐and‐Inclusion‐JEDI/DEI‐Activities. In parallel, publications by genetic counselors identified a persistent lack of diversity within NSGC’s membership and a need to prioritize diversity and inclusion (D&I) within the profession. This included addressing barriers to entry and collecting data to explore and support solutions.

In 2018, the NSGC Board of Directors included D&I as a strategic area of focus in its Strategic Plan, codifying it as a priority for the organization. A Task Force convened to explore this and future work, with efforts later expanding to include justice and equity and, thus, J.E.D.I. NSGC created a committee as a permanent group to continue these efforts. NSGC’s D&I Task Force recommended engaging an external DEI consultant to guide its efforts, and The Exeter Group (Exeter) was selected. Exeter undertook an organizational assessment for NSGC in 2020–2021, which included:

- Quantitative member data (from 4,583 current member profiles).
- Member and staff DEI survey (responses from 622 members and 24 staff).
- 25 focus groups (representing 22 diversity dimensions).
- 15 stakeholder interviews.
- Best practice research.
- Policy, process, and procedure review (63 documents).
- Benchmarking.

Exeter's completed assessment yielded several themes. Those relevant to the AGCPD Task Force's efforts described by Myers et al. were as follows: ‘Address Barriers to Entry to the Profession’, ‘Provide DEI Training and Resources’ and ‘Develop DEI Metrics and Communications’. Many under‐represented individuals shared concerns relevant to the ABGC certification process. Several described difficulties with taking the ABGC Board examination. Quotes within the report indicated potential Euro‐centric bias in ABGC certification examination questions, such as a bias toward individuals whose language of fluency is English. Others shared exclusionary training program experiences and feeling unwelcome as they entered the field, with calls for transformation and accountability.

The assessment identified needs and action steps to:

- Increase diversity within the genetic counseling field. Data from the member DEI survey show that 75% of respondents identified as White alone; 91% identified as women; 86% identified as heterosexual; 90% identified as having no disability; and 85% stated English as their primary language spoken.
- Address barriers and diversify pathways to entry including: low awareness of the field in communities with under‐represented individuals; training program requirements for admission; and the ABGC certification examinations.
- Develop DEI resources, metrics, and communications to build accountability. Limited collection of demographic data of NSGC volunteers and leaders hampers the understanding of diversity dimensions. There were also few to no DEI‐related continuing education opportunities and professional requirements.
- Collaborate on sustainable action to promote DEI throughout NSGC and the field. Grow and leverage partnerships across organizations to address potential bias in credentialing and certification examinations, as well as promote DEI in training programs.

The full DEI report and an Executive Summary are openly available on NSGC’s Web site, along with a video summarizing key findings of the report and the open comment period that followed (National Society of Genetic Counselors, 2021; NSGC, n.d.). NSGC’s J.E.D.I. webpage details the organization's activities up to the present time (Redlinger‐Grosse et al., 2020).

## 3 LOOKING TO THE FUTURE

After the publication of Exeter's DEI report, NSGC invited feedback from its members, non‐members, and individuals identifying within the genetics and genetic counseling communities. NSGC’s J.E.D.I. Committee summarized the feedback in a report to the Board of Directors in its Open Comment Period Board Memo (Redlinger‐Grosse et al., 2020). NSGC leadership also held calls with partner North American genetic counseling and genetics organizations to discuss collaboration opportunities. Myers et al. refer to the roles of four American organizations: NSGC (professional society), ABGC (certification board), ACGC (graduate program accrediting body), and AGCPD (graduate program directors association). Additional North American genetic counseling organizations include the Minority Genetics Professionals Network (MGPN), the Canadian Association of Genetic Counsellors (CAGC, Canadian professional society), and the Canadian Board of Genetic Counselling (CBGC, Canadian certification board). While these organizations have unique roles, these roles do not exist in a vacuum and all organizations must take responsibility for fostering greater diversity and inclusion in the field.

We wholeheartedly agree with the authors that collaboration among genetic counseling organizations is imperative, and a specific focus on this collaboration is articulated within the J.E.D.I. pillar of NSGC’s 2022–2024 Strategic Plan: https://www.nsgc.org/About/About‐NSGC/NSGC‐2022‐2024‐Strategic‐Plan. A new NSGC Task Force is developing a J.E.D.I. action plan for 2022 and beyond to delineate key steps to take across the organization, and in collaboration with partners. With this imperative in mind, informed by multiple data points including the work of Myers et al., Exeter's DEI report, and emergent themes from subsequent feedback and discussions with stakeholders, we propose four primary areas for collaboration: definitions and language alignment, data collection and sharing, resource development and sharing, and research into measuring quality genetic counseling in training and professional development.

### 3.1 Definitions and language alignment

Aligning on shared language and definitions related to J.E.D.I. work was a key need articulated in several of the discussions NSGC leadership held with leaders of other genetic counseling organizations. Given that we aim to increase diversity and inclusion at many intersecting points in the genetic counseling profession, it is critical to have a common lexicon by which to articulate and document goals and measure outcomes. Indeed, one of the prioritized recommendations from Exeter's report was for NSGC to ‘develop organization‐wide definitions of diversity, equity and inclusion’ (Exeter Group's DEI Report). We propose that these definitions should be aligned upon broadly throughout the profession.

### 3.2 Data collection and sharing

We propose that genetic counseling organizations commit to the collection and sharing of data related to recruitment and retention of genetic counselors representing diverse identities into the field. This is a necessary first step to identifying and assessing barriers and disparities in admissions guidelines, training, certification, ongoing education, and professional development. NSGC has made Exeter's DEI report publicly available and will continue to collect and share data through the Professional Status Survey and other means. We recognize and applaud genetic counseling training programs’ and organizations’ data collection and sharing efforts, an example being Myers et al.*’s* findings.

### 3.3 Resource development and sharing

We encourage the creation and sharing of resources for fostering greater justice, equity, diversity and inclusion in the training, supervising, education, mentoring, recruitment, and retention of genetic counselors. Such resources could include, for example, implicit bias and antiracism training, best practices for demographic data collection, and high school career fair outreach materials. We recognize that each genetic counseling organization has a unique purpose with specialty expertise and that access to monetary, volunteer, and staff assets vary. NSGC recognizes the need to bridge gaps and limitations to foster inclusive and equitable access by all partner organizations. Shared and easily accessible resources would maximize breadth and depth of content and eliminate duplicative efforts and redundancies, so that all genetic counselors could benefit and contribute to J.E.D.I. initiatives.

### 3.4 Research into measuring quality genetic counseling in training and professional development

Finally, we see the measurement of quality genetic counseling in training and professional development as an area for future research efforts and collaboration. Myers et al. suggest that collectively there could be broader inclusion of non‐cognitive variables such as self‐confidence, leadership, community service, critical thinking, and writing skills. Incorporating these qualities may help to redefine professional success. Using alternative assessment methods beyond the ABGC board examination could be considered to lead to better assessment of competence without bias. Furthering relationships with federal granting agencies such as the National Human Genome Research Institute could create avenues for filling existing research gaps in evidence‐based genetic counseling measurement.

## 4 CONCLUSION

Our goal in writing this response is to highlight and acknowledge the progress of our predecessors while recognizing the tall hill we have yet to climb. We also acknowledge that other healthcare professions with some parallels to genetic counseling have already explored similar issues in their respective fields (Wright et al., 2021; Moore n.d.) and are continuing these conversations, including evaluating novel outcomes‐based strategies for assessment (Harrison & Mitchell, 2006; Kak et al., 2001; Ryall et al., 2016; Sharpless, 2019). As a growing, nimble profession with the potential to learn and embrace a spirit of innovation, we can move toward outcome‐based competence for genetic counselors that can lead to a more just, equitable, diverse, and inclusive genetic counseling workforce. At a time of great upheaval in society, we are encouraged by our members’ support and energy toward NSGC’s and our profession's J.E.DI. efforts thus far. Continued, concerted efforts will need to be made to assess barriers to entry into the profession and how to adapt practice standards to reflect appropriate quality measures. We strongly encourage organizational collaboration in this collective work, with some concrete suggestions discussed earlier. Only by working together in a sustained and transparent way, and with accountability, will we make meaningful progress toward our shared vision of a profession that resonates with and delivers equitable care to the full range of people we serve.

## AUTHORS’ CONTRIBUTIONS

Heather Zierhut, Leila Jamal, Sara Riordan, Patrick Wilson, Meghan Carey, and Deepti Babu made substantial contributions to the conception of the Commentary and interpretation of the data from the referenced manuscript. Heather Zierhut, Leila Jamal, Sara Riordan, Patrick Wilson, Meghan Carey, and Deepti Babu contributed to the writing of the initial draft of the Commentary. All members of the Board of Directors and the Executive Director (Heather Zierhut, Leila Jamal, Sara Riordan, Patrick Wilson, Meghan Carey, Deepti Babu, Jodie Vento, Heather Hampel, Aishwarya Arjunan, Altovise Ewing, Carolyn Applegate, Barry Tong, and Sandy Prucka,) reviewed the Commentary, gave final approval of this version to be published, and were in agreement to be accountable for all aspects of the work in ensuring that questions related to the accuracy or integrity of any part of the work are appropriately investigated and resolved.

## CONFLICT OF INTEREST

Heather Zierhut researches genetic counseling skills, processes, and outcomes and owns stock in Genome Medical / GeneMatters, LLC.

Deepti Babu owns stock in ThinkGenetic, Inc., and is the founder of Integrity Content Consulting, a company that provides medical writing/content strategy services.

Sara Riordan owns stock or equity in Thermo Fisher Scientific, ixLayer and Unified Patient Network, and works with health systems and biopharma in genomic and clinical data sharing initiatives.

Heather Hampel is on the Scientific Advisory Board for Invitae Genetics, Genome Medical, and Promega. She has stock/stock options in Genome Medical and GI OnDemand.

Aishwarya Arjunan is an employee and shareholder of GRAIL/Illumina and also owns stock in Myriad Genetics.

Altovise Ewing is a full‐time employee of Roche/Genentech. She is on Medical Advisory Boards for The Chrysalis Initiative, Tulsa Innovations Lab (TIL) and Susan G. Komen's Stand for Health Equity Revolution Initiative‐ Genetic Counseling & Testing Advisory Group. She is a co‐founder of The Health Equity Solution and a paid consultant for the Genomes2People Consortium. She is a 23andMe and Roche shareholder.

Carolyn Applegate, Meghan Carey, Leila Jamal, Barry Tong, Sandy Prucka, Jodie Vento, and Patrick Wilson have no conflicts of interests to disclose.

**Classifications:** Diversity, Genetic Counseling, Diversity, Workforce.
